# The composite detoxification agent alleviates the toxicity induced by mycotoxins in Hy-Line Brown laying hens by regulating antioxidant capacity and gut bacterial communities

**DOI:** 10.1016/j.psj.2026.107347

**Published:** 2026-06-26

**Authors:** Yaling Ma, Qiaomei Gao, Junai Lu, Xianbai Liu

**Affiliations:** a Key Laboratory of Microbial Resources Exploitation and Application of Gansu Province, Institute of Biology, Gansu Academy of Sciences, Lanzhou, 730000, China; b Neiguanying Animal Husbandry and Veterinary Station, Anding District Animal Husbandry and Veterinary Bureau Dingxi, 743021, China; c State Key Laboratory of Grassland Agro-Ecosystems, Key Laboratory of Grassland Livestock Industry Innovation, Ministry of Agriculture and Rural Affairs, Engineering Research Center of Grassland Industry, Ministry of Education, Gansu Tech Innovation Centre of Western China Grassland Industry, College of Pastoral Agriculture Science and Technology, Lanzhou University, Lanzhou, 730020, China

**Keywords:** Composite detoxification agent, Laying performance, Serum biochemical indices, Antioxidant capacity, Gut microbiome, Laying hen

## Abstract

This study evaluated the efficacy of a composite detoxification agent in mitigating the adverse effects of naturally mold-contaminated feed in laying hens. A total of 800 Hy-Line Brown hens (156 days old) were randomly allocated to five dietary treatments (8 replicates with 20 birds per replicate), following a 7-d adaptation and a 113-d experimental period. The basal diet served as the control (CON group). Experimental treatments were structured as follows: ZH group (5% of normal corn in the feed replaced with moldy corn); ZJ group (5% of normal corn replaced with moldy corn + 0.1 g/kg composite detoxification agent); DH group (5% of normal soybean meal replaced with moldy cottonseed meal); DJ group (5% of normal soybean meal replaced with moldy cottonseed meal + 0.1 g/kg composite detoxification agent). Compared with CON, hens fed the ZH diet exhibited decreased (*P* < 0.05) egg production, average daily feed intake, egg weight, egg mass, and albumen quality, deteriorated feed conversion ratio. Serum biochemistry in ZH hens revealed lower total protein and alkaline phosphatase levels but higher blood urea nitrogen. Additionally, hens fed ZH diet displayed oxidative stress, characterized by elevated malondialdehyde (MDA) and reduced activities of total antioxidant capacity (T-AOC), catalase (CAT), superoxide dismutase (SOD), glutathione peroxidase (GSH-Px), glutathione S-transferase (GST), and nitric oxide (NO) (*P* < 0.05). Although less pronounced, similar alterations were observed in DH hens. Supplementation with the composite detoxifier restored laying performance and improved antioxidant status in both ZJ and DJ groups. Notably, the detoxifier enhanced gut microbiota diversity, enriched beneficial taxa including *Lactobacillus* and *Limosilactobacillus*, and correlated with alterations in the microbiota-host axis. These results indicate that the composite detoxifier alleviates mycotoxin-induced impairments and supports its application for managing feed mycotoxicosis in commercial layer production.

## Introduction

Mycotoxins are toxic secondary metabolites produced by filamentous fungi, particularly species of *Fusarium, Aspergillus*, and *Penicillium* (Gao et al., 2023). The Food and Agriculture Organization of the United Nations (FAO) estimates that approximately 25% of global agricultural commodities including cereals, oilseeds, feedstuffs, and medicinal herbs are contaminated with mycotoxins (Eskola et al., 2020). Such contamination results in annual economic losses amounting to hundreds of billions of US dollars and poses a severe threat to food and feed safety. Mycotoxins may persist on raw commodities as well as processed derivatives, exhibiting diverse toxicological properties, including hematopoietic suppression, carcinogenicity, mutagenicity, teratogenicity, immunosuppression, and disruption of normal growth and development in both humans and animals (Su et al., 2021; Sun et al., 2023). The major classes of mycotoxins include aflatoxins (AFs), zearalenone (ZEA), deoxynivalenol (DON), ochratoxin A (OTA), T-2 toxin, fumonisins (FUMs), and patulin (PAT). Collectively, mycotoxins represent a critical public health concern and a significant impediment to global food security (Sohrabi et al., 2022).

In poultry production, mycotoxin-contaminated feedstuffs significantly compromise health and productivity, leading to substantial economic losses (Winter and Pereg, 2019). Common mycotoxins such as AFs, FUMs, and ZEA impair growth, reduce egg production, degrade egg quality, and induce oxidative stress and organ damage (Zhu et al., 2023). Naturally contaminated feed often contains multiple mycotoxins; thus, evaluating their combined toxicological and physiological effects is essential (Christofidou et al., 2015). Although physical (e.g., adsorbents), biological (e.g., probiotics and biotransformation enzymes), and antioxidant-based strategies are widely employed to alleviate mycotoxin toxicity (Jia et al., 2025), single-strategy interventions frequently prove inadequate against complex co-contamination profiles. To overcome these limitations, efforts have focused on integrating multiple mitigation strategies into unified systems. Consequently, composite detoxification agents that integrate clay- or yeast-derived adsorbents, probiotic strains, biotransformation enzymes, and antioxidants have gained increasing attention as a synergistic mitigation approach (Eratak et al., 2023; Nadya et al., 2025).

Such formulations are rationally designed based on three complementary mechanisms: probiotic adsorbents physically reduce gastrointestinal absorption of mycotoxins (Ndiaye et al., 2022); specific biotransformation enzymes catalyze the degradation of toxin structures into less harmful metabolites (Ben Taheur et al., 2019); and antioxidants alleviate oxidative injury induced by residual toxins (Deníz et al., 2025; Nadya et al., 2025). Compared with single-strategy interventions, this integrated approach has demonstrated superior efficacy in restoring physiological homeostasis and improving production performance in poultry (Guo et al., 2023).

Against this background, the present study evaluated the effects of composite detoxification agents on laying hens fed diets contaminated with moldy corn or moldy cottonseed meal. The study focused on key parameters including production performance, egg quality, serum biochemistry, antioxidant status, and gut microbiota. This study provides a theoretical basis and practical evidence for the application of composite detoxification agents to mitigate feed mycotoxicosis and enhance the health and productivity of commercial laying hens.

## Materials and methods

### Diets

To prepare the moldy corn and cottonseed meal, raw materials were covered with plastic film and insulating pads. Water was sprayed weekly, and the materials were stirred thoroughly to ensure even moisture distribution. Over a 30-d period, spontaneous molding occurred under natural conditions (temperature: 14-20 °C; relative humidity: 65-70%). After molding, mycotoxin profiles were determined using a fluorescent quantitative rapid detector (LD-L02, Shandong Haiman Scientific Instrument Co., Ltd.). The analyzed contents of aflatoxins B₁ (AFB₁), DON, ZEN, fumonisins B₁ (FB₁), OTA, and T-2 toxin are summarized in Table S1 (Liu et al., 2024; Zhang et al., 2022). The moldy corn was characterized by high levels of FB₁ (48964.04 *μ*g/kg) and moderate levels of AFB₁ (80.83 *μ*g/kg) and ZEN (148.36 *μ*g/kg). Conversely, the moldy cottonseed meal exhibited notably high concentrations of FB₁ (26974.25 *μ*g/kg), along with detectable AFB₁ (78.09 *μ*g/kg) and ZEN (602.17 *μ*g/kg). In both ingredients, DON remained below the regulatory limit, whereas AFB₁ and FB₁ exceeded the national standard (GB 13078-2017). Neither OTA nor T-2 toxin was detected in either sample.

### Experimental design

A total of 800 Hy-Line Brown laying hens aged 156 days were housed in stepped cages and randomly allocated into 5 groups using a randomized block design. Each group included 8 replicates, with 20 birds per replicate. After a 7 day adaptation period, the formal trial lasted 113 days. In each group, three birds were individually housed in cages measuring (0.47 × 0.37 × 0.38 m). The basal diet served as the control (CON group). Experimental treatments were structured as follows: ZH group (5% of normal corn in the feed replaced with moldy corn); ZJ group (5% of normal corn replaced with moldy corn + 0.1 g/kg composite detoxification agent); DH group (5% of normal soybean meal replaced with moldy cottonseed meal); DJ group (5% of normal soybean meal replaced with moldy cottonseed meal + 0.1 g/kg composite detoxification agent). The 5% replacement level of moldy ingredients was selected to simulate realistic on-farm contamination scenarios, in which inadvertent inclusion of low-level moldy feedstuffs is common in commercial layer production (Zhang et al., 2022; Zhu et al., 2023). The composition and nutrient levels of the basal diet are shown in Table S2. The composite detoxification agent consisted of three functionally complementary components: *Lactobacillus plantarum* for enzymatic degradation of mycotoxins, mannan-oligosaccharides (MOS) for competitive adsorption and gut microbiota modulation, and montmorillonite for adsorption of residual toxins. The detailed formula is provided in Table S3. During the experimental period, the laying hens were provided with unrestricted access to feed and water, with feeding facilitated by a mechanical feeder and watering through nipple type drinkers. The light cycle was 16 h light: 8 h darkness, with two daily feedings at 08:00 and 14:00. Eggs were collected manually at 09:00. Routine sanitation of the poultry house and scheduled vaccinations were conducted regularly. All experimental procedures were reviewed and approved by the Institutional Animal Care and Use Committee (IACUC) of Lanzhou University (Approval No. LZU20230920).

### Fecal sample collection and pretreatment

Three days prior to the end of the experiment, six hens per replicate were randomly selected for fecal collection. Fresh feces were collected daily over three consecutive days, and approximately 50 g of fresh feces per bird per day was weighed and immediately stored at −20°C. Prior to lyophilization, fecal samples were thawed at 4°C overnight, weighed to determine the initial wet weight, and then transferred to a pre‑cooled freeze dryer (LGJ‑25 G; Sihuan Furuike Technology Development Co., Ltd.) operating at −66°C. Lyophilization was conducted for approximately 48 h until a constant weight was achieved. The dried samples were weighed again to calculate dry matter content, ground through a 0.5 mm sieve, and stored at −20°C until mycotoxin residue analysis. All mycotoxin concentrations in feces are expressed on a dry matter (DM) basis.

### Determination of feed nutrient composition

The methods for determining the crude protein, calcium, and total phosphorus content in feed were carried out in accordance with the national standard methods, respectively. Among them, the protein detection was based on GB/T 6432-2018, calcium detection based on GB/T 6436-2018, total phosphorus detection based on GB/T 6437-2018, and metabolizable energy was calculated according to the ‘Table of Feed Composition and Nutritional Values of China’ (33rd Edition, 2022).

### Measurement of mycotoxins

The determination of mycotoxins in feed and fecal samples was carried out using a rapid detection instrument for mycotoxins by fluorescent quantitative detection (LD-L02, Shandong Haiman Scientific Instrument Co., Ltd.). This method was used to determine the content of common mycotoxins AFB₁, ZEN, DON, FB_1_, OTA, and T-2 in both feed and fecal samples. The instrument utilizes a competitive inhibition immunoassay principle. Prior to sample analysis, the instrument was calibrated using a series of standard solutions provided by the manufacturer to establish standard curves (*R^2^*>0.99). The limits of detection (LOD) and limits of quantification (LOQ) for the assay were as follows: AFB₁ (LOD: 4 *μ*g/kg, LOQ: 100 *μ*g/kg), ZEN (LOD: 10 *μ*g/kg, LOQ: 1000 *μ*g/kg), DON (LOD: 100 *μ*g/kg, LOQ: 5000 *μ*g/kg), FB₁ (LOD: 100 *μ*g/kg, LOQ: 5000 *μ*g/kg), OTA (LOD: 4 *μ*g/kg, LOQ: 1000 *μ*g/kg), and T-2 toxin (LOD: 10 *μ*g/kg, LOQ: 5000 *μ*g/kg). Sample preparation involved homogenization and extraction according to the manufacturer’s protocol. The regulatory limits for mycotoxins were referenced to the Chinese Feed Hygiene Standard (GB 13078-2017) (General Administration of Quality Supervision, 2017; Zhao et al., 2024).

### Production performance

During the trial period, the number of eggs laying, egg weight, feed given, and leftover feed were counted for each replicate. The egg laying rate, average egg weight, egg mass, average daily feed intake, and feed to egg ratio of each group of laying hens were then calculated.

### Egg quality

At the end of the experiment, 30 eggs were randomly selected from each replicate per group. The transverse and longitudinal diameters of the eggs were measured using a vernier caliper (N15P, Mitutoyo Precision Measuring Instruments Co., Ltd.), and the egg shape index was calculated as the ratio of longitudinal diameter to transverse diameter. The shell thickness at the pointed end, middle, and blunt end of the eggs was measured using an eggshell thickness gauge (NFN380, Beijing Brad Technology Development Co., Ltd.), and the average of the three measurements was taken as the eggshell thickness. Eggshell strength was determined using an automatic eggshell strength tester (KQ-1A, Beijing Tianxiang Feiyu Testing Equipment Co., Ltd.). The height of the egg white was detected using an egg quality tester (YN-11 L, Nanjing Yao'en Instrument Equipment Co., Ltd.). The Haugh units were calculated using the formula: 100 × log_10_ [albumen height + 7.57 - 1.7 × (egg weight ^0.37^)]. Subsequently, the egg white and chalaza were separated, and the yolk weight was measured using an electronic balance. Yolk percentage was calculated as: Yolk percentage (%) = 100 × (yolk weight / egg weight) (Gao et al., 2021).

### Analysis of biochemistry and antioxidant indices in Serum

At the official end of the experiment, six laying hens were randomly selected from each replicate per group. Ten milliliters of blood was collected using the wing vein blood sampling method and injected into a coagulant tube (Guo et al., 2021). After standing for 30 minutes to allow the blood to fully clot, the tube was placed in a centrifuge and centrifuged at 2000 × g for 10 minutes. The upper clear serum was collected and transferred to a 1.5 mL LEP tube, labeled properly, and stored at −20 °C. Commercial kits (Suzhou Yanxi Biotechnology Co., Ltd, Suzhou, China) were used to measure the activities of total protein (TP; cat. no. A040-1-1), albumin (ALB; cat. no. A041-1-2), alanine aminotransferase (ALT; cat. no. C008-3), aspartate aminotransferase (AST; cat. no. C09-2-2), alkaline phosphatase (ALP; cat. no. A035-2), urea nitrogen (BUN; cat. no. A036-1), total antioxidant capacity (T-AOC; cat. no. A021-1-1), catalase (CAT; cat. no. A014-1-2), superoxide dismutase (SOD; cat. no. A010-2-1), glutathione peroxidase (GSH-Px; cat. no. A003-3-1), glutathione S-transferase (GST; cat. no. A010-2-1), malondialdehyde (MDA; cat. no. A015-3-1), nitric oxide (NO; cat. no. A016-2-1) were detected spectrophotometrically according to manufacturer’s protocol.

*DNA extraction*

At the end of the experimental period, after a 12 hour fast, six laying hens with similar average weights were randomly selected from each replication per group for slaughter. After slaughter, the jejunum was quickly removed from each hen, and the contents were collected in sterile containers. They were then quickly frozen with liquid nitrogen and stored at −80°C for subsequent microbial community sequencing (Sihem et al., 2025). Total DNA from all samples were extracted using the MN NucleoSpin 96 Soil DNA kit (MACHEREY-NAGEL, Düren, Germany). DNA quantity and quality were measured on a NanoDrop 2000 spectrophotometer (Thermo Fisher Scientific (China) Co., Ltd., China). The total DNA was stored in a laboratory freezer at −80°C until use.

### Sequencing and analysis of 16S rRNA gene

High-throughput sequencing of 16S rRNA were employed to analyze the gut microbial community structure. The operational process, which included jejunum contents DNA extraction, amplification, library construction, sequencing, and data analysis, was conducted by Biomarker Technologies Corporation, Beijing, China. Full-length gut bacterial 16S rRNA were amplified using barcode-conserved bacterial primers 27F (5′-AGAGTTTGATCCTGGCTCAG-3′) and 1492R (5′-GGTTACCTTGTTACGACTT-3′). The amplified library was sequenced on a PacBio SMRT RS II platform (Pacific Biosciences, Menlo Park, CA, USA). The Raw data (paired end reads) were assembled and filtered using Lima v1.7.0, and chimeras were removed using UCHIME v4.2 (Effective Tags). Key metrics calculated included average length, GC content, Q20 (proportion of bases with quality value > 20), Q30 (proportion of bases with quality value > 30), and effective ratio. Classification (Zheng et al., 2018) and statistical analysis (Bokulich et al., 2013) were performed as previously described. This enabled identification of the ‘core microbiome’ for each group and the ‘core gut microbiome’ across all samples. Bacterial community indices used included Chao1, Ace, and Shannon. Alpha and beta diversity were estimated using the Bray-Curtis distance measure (via Mothur v.1.30 and QIIME). Principal component analysis (PCA) of weighted UniFrac distance matrices was used for clustering. Linear discriminant analysis (LDA) was performed using BMK Cloud to identify statistically significant differences between treatments, with LDA scores exceeding 4. Redundancy analysis (RDA) was conducted to examine relationships between environmental characteristics and gut bacterial community composition. Phylogenetic Investigation of Communities by Reconstruction of Unobserved States (PICRUSt) analysis (http://picrust.github.io/picrust/) was used to predict the effects of different treatments on the laying hen gut bacterial community (Liu et al., 2020).

### Data analysis

Data were analyzed using SPSS 21.0 (SPSS, Chicago, IL, USA), and one-way analysis of variance (ANOVA) was conducted, followed by Tukey’s post hoc test for multiple comparisons. Results are reported as mean ± standard error (SE), with statistical significance defined as *P* < 0.05. Additionally, the alpha diversity of the laying hen gut bacterial community, influenced by different treatments, was analyzed using the Mantel test based on Spearman’s product-moment correlation.

## Results

### Contents of mycotoxins in the experimental diets

The analyzed contents of mycotoxins in the experimental diets are summarized in Table S4. The control group diet contained AFB₁ 4.89 *μ*g/kg and ZEN 10.85 *μ*g/kg, both of which were below the limits specified in the Chinese Feed Hygiene Standard (GB, 13078‑2017); DON, FB₁, OTA, and T-2 toxin were not detected. The ZH group diet contained AFB₁ 13.94 *μ*g/kg, ZEN 68.36 *μ*g/kg, and FB₁ 14385.65 *μ*g/kg; the ZJ diet contained AFB₁ 6.16 *μ*g/kg, ZEN 21.09 *μ*g/kg, and FB₁ 7601.02 *μ*g/kg; the DH group diet contained AFB₁ 8.91 *μ*g/kg, DON 141.27 *μ*g/kg, ZEN 93.42 *μ*g/kg, and FB₁ 13844.39 *μ*g/kg; the DJ group diet contained AFB₁ 5.75 *μ*g/kg, ZEN 40.43 *μ*g/kg, and FB₁ 6335.44 *μ*g/kg. OTA and T-2 toxin were not detected in any treatment diet.

### Fecal mycotoxin residues and detoxification efficiency

Fecal mycotoxin residues are summarized in Table S5. Due to the assay’s detection limits, AFB₁ was not quantifiable (<4 *μ*g/kg) in the CON, ZJ, and DJ groups, whereas it was quantifiable at 12.44 *μ*g/kg in the ZH group and 8.04 *μ*g/kg in the DH group. Similarly, ZEN was below the LOD (<10 *μ*g/kg) in the CON group but detectable in the mycotoxin-challenged groups (ZH: 64.19 *μ*g/kg; ZJ: 14.91 *μ*g/kg; DH: 90.21 *μ*g/kg; DJ: 32.98 *μ*g/kg). Notably, the concentration of extractable FB₁ in the ZJ (936.56 *μ*g/kg) and DJ (1117.53 *μ*g/kg) groups were markedly lower than that in their respective positive control groups (ZH: 12345.74 *μ*g/kg; DH: 13377.18 *μ*g/kg). DON was detected only in the DH group (132.44 *μ*g/kg), which is consistent with the dietary contamination level (Table S4). OTA and T-2 toxin were not detected in any fecal sample.

### Effects of composite detoxification agent on the performance of laying hens

Supplementation with the composite detoxification agent significantly affected (*P* < 0.05) the egg laying rate, average daily feed intake, average egg weight, egg mass, and feed to egg ratio (Table 1), albumen height, and Haugh unit (Table 2) in laying hens. Compared with the control group (CON), the ZH diet significantly reduced the laying rate (82.14% vs 95.59%), average daily feed intake (113.51 g vs 130.64 g), average egg weight (57.31 g vs 63.11 g), and egg mass (47.08 g vs 60.39 g), and feed to egg ratio (1.98 vs 2.07) (Table 1). Upon the incorporation of the composite detoxification agent into the ZJ group, egg laying rate observed indicators reverted to the levels equivalent to those in the control group. In comparison to the DJ group, the DH group exhibited a decrease in the egg laying rate, average daily feed intake, the average egg weight and egg mass, whereas the DJ group supplemented with the composite detoxification agent demonstrated enhanced performance in these indicators (Table 1). The ZH group displayed significantly lower albumen height and Haugh unit values in comparison to the CON, ZJ, DH, and DJ groups, whereas the ZJ and DJ groups supplemented with the composite detoxification agent exhibited improved effects, restoring restored these parameters to levels comparable to those of the CON group. Other egg quality parameters were not significantly affected (*P* > 0.05; Table 2).

**Table 1:** Effects of composite detoxification agent on the production performance of laying hens.

Items	CON	ZH	ZJ	DH	DJ	*P*-value
Egg laying rate (%)	95.59±2.47^a^	82.14±3.23^b^	89.22±2.91^ab^	91.96±2.48^ab^	94.64±2.08^a^	0.004
Average daily feed intake (g)	130.64±0.62^a^	113.51±3.23^b^	123.73±1.42^a^	127.19±0.74^a^	129.54±0.91^a^	<0.001
Average egg weight (g)	63.11±0.26^a^	57.31±0.11^d^	59.19±0.26^c^	59.48±0.21^c^	61.50±0.09^b^	<0.001
Egg mass (g)	60.39±1.62^a^	47.08±1.86^c^	53.29±1.63^bc^	54.70±1.49^ab^	58.19±1.28^ab^	<0.001
Feed to egg ratio	2.07±0.01^ab^	1.98±0.06^b^	2.09±0.03^ab^	2.14±0.02^a^	2.11±0.02^ab^	0.006

CON: basic diet, ZH: 5% of normal corn in the feed replaced with moldy corn, ZJ: 5% of normal corn replaced with moldy corn + 0.1 g/kg composite detoxification agent, DH: 5% of normal soybean meal replaced with moldy cottonseed meal, DJ: 5% of normal soybean meal replaced with moldy cottonseed meal + 0.1 g/kg composite detoxification agent.

Means with no superscripts within a row are no signiffcantly different (*P* > 0.05), Means with different superscripts(a,b) within a row are signiffcantly different (*P* < 0.05). The same as below.

**Table 2:** Effects of composite detoxification agent on the egg quality of laying hens.

Items	CON	ZH	ZJ	DH	DJ	*P*-value
Egg shape index	1.12±0.03	1.20±0.06	1.17±0.04	1.13±0.03	1.23±0.04	0.245
Shell thickness (mm)	40.38±0.84	39.37±0.45	40.27±0.61	39.07±0.33	40.33±0.27	0.286
Eggshell strength (kg/cm^2^)	0.51±1.85	0.47±1.71	0.54±1.90	0.49±2.09	0.53±1.84	0.102
Albumen height (mm)	7.58±0.18^a^	6.09±0.29^b^	7.40±0.28^a^	7.15±0.17^a^	7.57±0.21^a^	<0.001
Haugh unit	87.08±0.99^a^	77.81±1.99^b^	85.97±1.52^ab^	83.37±1.16^ab^	86.47±1.06^ab^	<0.001
Yolk color	10.30±0.15	10.30±0.30	10.80±0.20	10.30±0.15	10.60±0.16	0.283
Yolk weight (g)	15.82±0.28	14.53±0.22	14.56±0.41	14.59±0.59	15.48±0.33	0.059
Yolk ratio (%)	25.07±0.36	25.35±0.66	24.60±0.71	24.53±0.48	25.17±0.62	0.732

CON: basic diet, ZH: 5% of normal corn in the feed replaced with moldy corn, ZJ: 5% of normal corn replaced with moldy corn + 0.1 g/kg composite detoxification agent, DH: 5% of normal soybean meal replaced with moldy cottonseed meal, DJ: 5% of normal soybean meal replaced with moldy cottonseed meal + 0.1 g/kg composite detoxification agent.

### Effects of composite detoxification agent on serum biochemical and antioxidant status of laying hens

Supplementation with the composite detoxification agent significantly affected (*P* < 0.05) the serum biochemical indices in laying hens (*P* < 0.05; Table 3). Compared with the CON group, the ZH group had lower serum TP and higher BUN. The ZJ and DJ group with the addition of the composite detoxification agent showed ameliorative effects. Supplementation with the composite detoxification agent significantly improved the antioxidant status of laying hens (*P* < 0.05; Table 4). Moldy feeds induced oxidative stress, significantly decreasing T-AOC, CAT, SOD, GSH-Px, GST, NO and increasing MDA, while the ZJ and DJ group with the addition of the composite detoxification agent showed significantly improved T-AOC, CAT, SOD, GSH-Px, GST and reduced MDA across groups (Table 4).

**Table 3:** Effects of composite detoxification agent on serum biochemical indices of laying hens.

Items	CON	ZH	ZJ	DH	DJ	*P*-value
Total protein, TP (mg/mL)	64.46±0.25^a^	54.49±0.58^b^	56.34±0.22^ab^	60.04±0.04^ab^	64.66±0.24^a^	<0.001
Albumin, ALB(mg/mL)	18.59±0.26^b^	17.44±0.24^b^	18.72±0.29^b^	17.97±0.27^b^	19.46±0.25^a^	0.003
Alanine aminotransferase, ALT(nmol/min/mg prot)	5.68±0.17^b^	4.92±0.16^b^	6.53±0.12^a^	5.79±0.28^b^	6.15±0.15^a^	0.001
Aspartate aminotransferase, AST(nmol/min/mg prot)	6.14±0.12^b^	5.27±0.17^a^	7.01±0.29^a^	6.19±0.12^ab^	6.60±0.23^a^	0.001
Alkaline phosphatase, ALP (nmol/min/mg prot)	0.92±0.23^b^	0.43±0.12^c^	1.11±0.99^a^	0.56±0.31^bc^	0.81±0.11^b^	<0.001
Urea nitrogen, BUN (mg/mL)	0.07±0.001^b^	0.10±0.001^a^	0.08±0.003^a^	0.08±0.002^a^	0.08±0.001^a^	<0.001

CON: basic diet, ZH: 5% of normal corn in the feed replaced with moldy corn, ZJ: 5% of normal corn replaced with moldy corn + 0.1 g/kg composite detoxification agent, DH: 5% of normal soybean meal replaced with moldy cottonseed meal, DJ: 5% of normal soybean meal replaced with moldy cottonseed meal + 0.1 g/kg composite detoxification agent.

**Table 4:** Effects of composite detoxification agent on serum antioxidant indices of laying hens.

Items	CON	ZH	ZJ	DH	DJ	*P*-value
Total antioxidant capacity, T-AOC (μmol Trolox/mL)	0.21±0.011^a^	0.17±0.002^b^	0.25±0.001^a^	0.17±0.001^b^	0.23±0.001^a^	<0.001
Catalase, CAT (μmol/min/mg prot)	0.60±0.12^a^	0.43±0.004^b^	0.70±0.01^a^	0.34±0.01^c^	0.48±0.01^b^	<0.001
Superoxide dismutase, SOD (U/mg prot)	2.41±0.11^a^	1.41±0.03^c^	2.09±0.01^b^	1.68±0.01^bc^	2.17±0.06^b^	<0.001
Glutathione peroxidase, GSH-Px (nmol/min/mg prot)	13.82±0.22^a^	9.22±0.03^b^	14.21±0.12^a^	13.05±0.03^ab^	13.53±0.29^a^	<0.001
glutathione S-transferase, GST (nmol/min/mg prot)	2.53±0.02^a^	1.93±0.003^c^	2.37±0.04^b^	1.86±0.003^d^	2.30±0.003^b^	<0.001
Nitric oxide, NO (μmol/mL)	0.02±0.001^a^	0.01±0.0009^b^	0.01±0.006^c^	0.01±0.0006^b^	0.01±0.003^c^	<0.001
Malondialdehyde, MDA (nmol/mL)	3.48±0.01^d^	8.48±0.25^a^	5.02±0.03^b^	4.18±0.06^c^	2.82±0.02^d^	<0.001

CON: basic diet, ZH: 5% of normal corn in the feed replaced with moldy corn, ZJ: 5% of normal corn replaced with moldy corn + 0.1 g/kg composite detoxification agent, DH: 5% of normal soybean meal replaced with moldy cottonseed meal, DJ: 5% of normal soybean meal replaced with moldy cottonseed meal + 0.1 g/kg composite detoxification agent.

### Analysis of 16S rRNA Sequencing

**Fig. 1:**
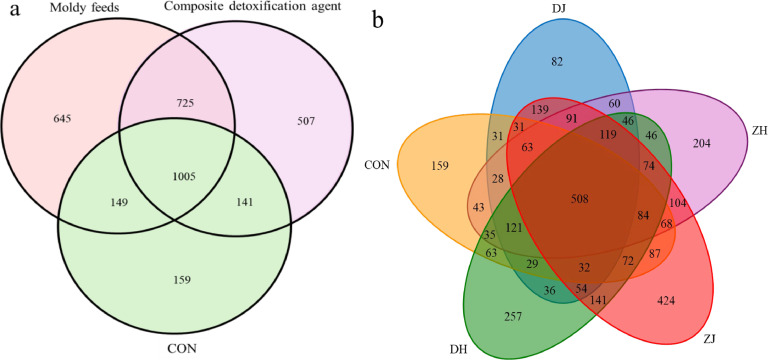
(a) Venn diagram illustrating the operational taxonomic units (OTUs) unique to each group of laying hens: Moldy feed (including ZH and DH), Composite detoxification agent (including ZJ and DJ), and Control (CON). (b) Overlap of OTUs among the gut microbial communities of laying hens under different treatments.

**Fig. 2:**
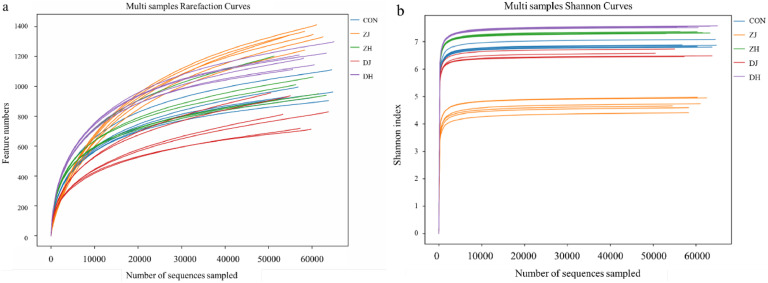
(a) The sample rarefaction curves and (b) the Shannon index rarefaction curves. CON: basic diet, ZH: 5% of normal corn in the feed replaced with moldy corn, ZJ: 5% of normal corn replaced with moldy corn + 0.1 g/kg composite detoxification agent, DH: 5% of normal soybean meal replaced with moldy cottonseed meal, DJ: 5% of normal soybean meal replaced with moldy cottonseed meal + 0.1 g/kg composite detoxification agent.

Microbial community compositions of 30 gut samples from five groups were obtained by PacBio sequencing. Amplicons were sequenced and identified by barcode, a total of 1836813 CCS (Circular Consensus Sequencing) sequences were obtained. Each sample produced at least 54003 CCS sequences; with an average of 61227 CCS sequences (Table S6). Cluster analysis at 97% sequence similarity levels, obtained a total of 3331 OTUs (Fig. 1a), as shown in the Venn diagrams, there were 645 OTUs that were identified in moldy feeds samples, 507 OTUs that were identified in composite detoxification agent, 159 OTUs that were identified in CON (Fig. 1a). One thousand four hundred fifty-four OTUs were detected in CON samples, 1470 OTUs were detected in DJ samples, 1694 OTUs were detected in ZH samples, 2091 OTUs were detected in ZJ samples, and 1717 OTUs were detected in DH samples, and 508 “core” OTUs were present in samples from all different treatments (Fig. 1b), including 37 phylum, 82 classes, 209 orders, 397 families, 918 genus, and 1620 species (Table S7). The sample rarefaction curve (Fig. 2a), and Shannon index rarefaction curve (Fig. 2b) show that the sequencing volume is sufficient, and the sequencing depth is saturated, and increasing the sample volume that will not produce more OTUs. Meanwhile, the completeness of sequencing we used Good’s coverage to check. The results showed that coverage was > 98% each sample, indicating that most species in sample have been identified.

### Comparison of the Gut Microbiota

Alpha diversity reflects changes in the gut bacterial between and within each species (Table 5). In general, gut bacterial communities associated with composite detoxification agent were significantly (*P* < 0.05). ZJ had the highest Chao and ACE richness values, and DH had the highest Shannon and Simpson diversity indices, whereas DH and ZJ held the lowest bacterial richness and diversity, respectively. There was significant difference in composite detoxification agent between Chao1, Shannon, Simpson and ACE. Therefore, the diversity of gut bacterial community in laying hens was relatively correlated with the content of the composite detoxification agent.

**Table 5:** The richness of gut bacterial communities of laying hens.

Items	CON	ZH	ZJ	DH	DJ	*P*-value
Chao1	1135.21±50.26^b^	1237.24±61.45^b^	1598.93±34.95^a^	1053.79±74.73^b^	1354.92±34.19^b^	<0.001
Shannon	6.89±0.04^b^	7.37±0.04^a^	4.72±0.09^d^	7.55±0.01^a^	6.53±0.04^c^	<0.001
Simpson	0.97±0.0006^a^	0.98±0.0005^a^	0.84±0.008^b^	0.99±0.0001^a^	0.97±0.0007^a^	<0.001
ACE	1099.82±41.84^b^	1216.44±63.83^b^	1595.73±32.02^a^	1046.42±66.04^b^	1330.08±35.42^a^	<0.001

CON: basic diet, ZH: 5% of normal corn in the feed replaced with moldy corn, ZJ: 5% of normal corn replaced with moldy corn + 0.1 g/kg composite detoxification agent, DH: 5% of normal soybean meal replaced with moldy cottonseed meal, DJ: 5% of normal soybean meal replaced with moldy cottonseed meal + 0.1 g/kg composite detoxification agent.

Different lowercase letters in a row of data indicate significant differences at *P* < 0.05 among different treatments.

**Fig. 3:**
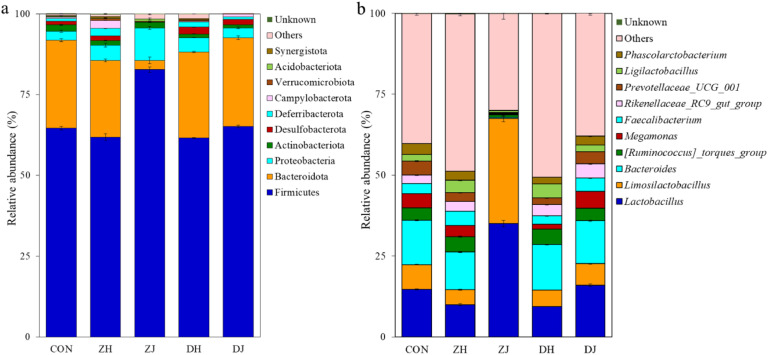
The top 10 relative abundances of gut bacterial community at phylum level (a) and genus level (b). Each color indicate a species, the height of the color block represents the proportion of species in relative abundance. The other species shown in Fig. are merged into “other”. “Unknown” indicates species that there are not received a taxonomic annotation. CON: basic diet, ZH: 5% of normal corn in the feed replaced with moldy corn, ZJ: 5% of normal corn replaced with moldy corn + 0.1 g/kg composite detoxification agent, DH: 5% of normal soybean meal replaced with moldy cottonseed meal, DJ: 5% of normal soybean meal replaced with moldy cottonseed meal + 0.1 g/kg composite detoxification agent.

The gut bacterial community was identified at different taxonomic levels. At the phylum level (Fig. 3a), Firmicutes, Bacteroidota, Proteobacteria, Actinobacteriota, Desulfobacterota, Deferribacterota, Campylobacterota, Verrucomicrobiota, Acidobacteriota, and Synergistota were the top 10 phyla in the relative abundance. Among them, Firmicutes was the absolutely dominant phylum, and the ZJ group had the highest relative abundance of 82.79 ± 0.83%. The relative abundances in the other groups were as follows: 65.15 ± 0.39% in DJ, 64.60 ± 0.57% in CON, 61.86 ± 0.98% in ZH, 61.57 ± 0.12% in DH. Bacteroidota was the top abundance bacteria in DJ, CON, and ZH, and the relative abundances were 27.48 ± 0.53%, 27.26 ± 0.46%, 23.68 ± 0.44%, respectively. Proteobacteria was also dominant in the ZJ, ZH, DH, DJ and CON the relative abundances were 10.09 ± 0.48%, 4.78 ± 0.65%, 4.47 ± 0.08%, 2.99 ± 0.28% and 2.72 ± 0.34%, respectively. At the family level (Fig. S1), Lactobacillaceae dominant in the ZJ the relative abundances were 68.26 ± 0.93%, respectively. Lachnospiraceae was dominant in the DJ, and the relative abundances was 13.81 ± 0.21%. Bacteroidaceae was dominant in the DH, and the relative abundances was 13.99 ± 0.08%. At the genus level (Fig. 3b) of *Lactobacillus* and *Limosilactobacillus* were the main component of gut bacterial community of laying hen in the ZJ group having the highest relative abundance 35.00 ± 0.96% and 32.48 ± 1.00%. *Bacteroides* was the main component of gut bacterial community of composite detoxification agent laying hen in the DH, CON and DJ, and the relative abundance of 13.99 ± 0.08%, 13.70 ± 0.15%, and 13.28 ± 0.21%.

**Fig. 4:**
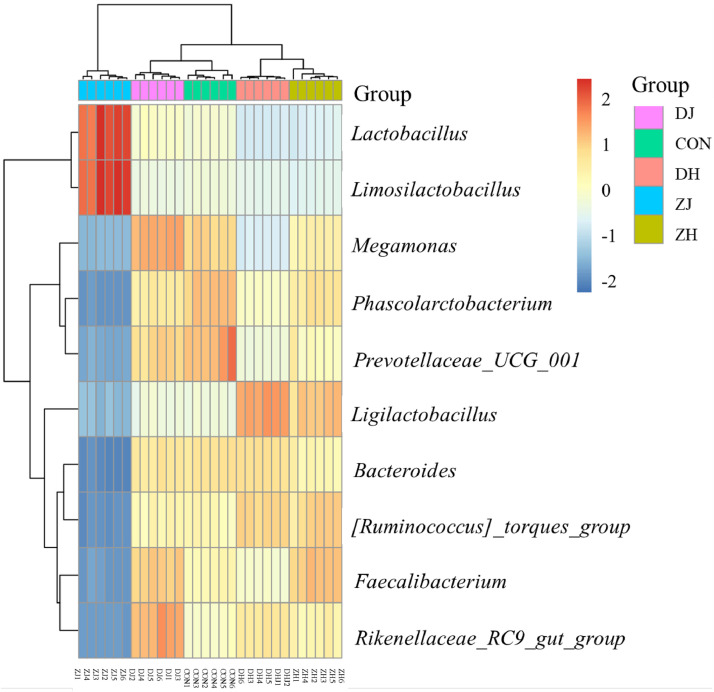
Heatmap of the top 20 abundant genera in the bacterial communities illustrating the average abundances of bacterial taxa assigned to the genus level. The columns represent the samples, and the row represents the bacterial assigned to species level. Dendrograms of hierarchical cluster analysis of taxonomic genera and samples were displayed on the left and top, respectively. The color scale used log 10 to represent the standardized values of relative abundances. CON: basic diet, ZH: 5% of normal corn in the feed replaced with moldy corn, ZJ: 5% of normal corn replaced with moldy corn + 0.1 g/kg composite detoxification agent, DH: 5% of normal soybean meal replaced with moldy cottonseed meal, DJ: 5% of normal soybean meal replaced with moldy cottonseed meal + 0.1 g/kg composite detoxification agent.

To visualize the dynamic pattern of bacterial communities in the intestines of laying hens, a heatmap was generated based on the dataset obtained from laying hen (Fig. 4). As the study focused on abundant taxa, the 20 genera with the highest relative abundances were selected for this analysis. Clustering was performed based on species abundance similarity, where horizontal clustering representing sample information and vertical clustering representing species information.

**Fig. 5:**
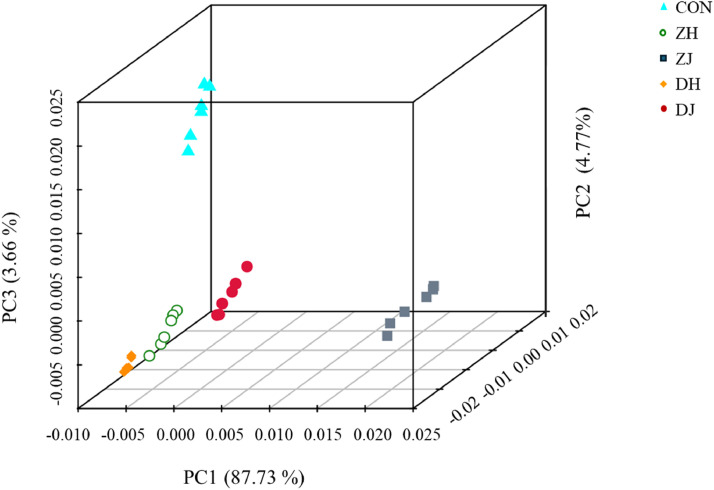
Principal coordinate analysis (PCoA) of laying hens gut bacterial communities at the level of the operational taxonomic units (OTUs) based on Bray-Curtis analysis of different treatments. The percent variation explained by principal coordinate 1 (PC1) was 87.73%, whereas that explained by PC2 was 3.66% and 4.77%. CON: basic diet, ZH: 5% of normal corn in the feed replaced with moldy corn, ZJ: 5% of normal corn replaced with moldy corn + 0.1 g/kg composite detoxification agent, DH: 5% of normal soybean meal replaced with moldy cottonseed meal, DJ: 5% of normal soybean meal replaced with moldy cottonseed meal + 0.1 g/kg composite detoxification agent.

The Bray-Curtis dissimilarity principal coordinate analysis (PCoA) was employed to compare the differences in community composition among the samples. The PCoA scatter plot displays the first two principal coordinates, which account for the greatest variation between samples, explaining 87.73%, 3.66% and 4.77% of the variance, respectively (Fig. 5). The results of the PERMANOVA analysis indicated significant differences in the community structures among the various groups (PERMANOVA: R² = 0.764, *P* = 0.001).

**Fig. 6:**
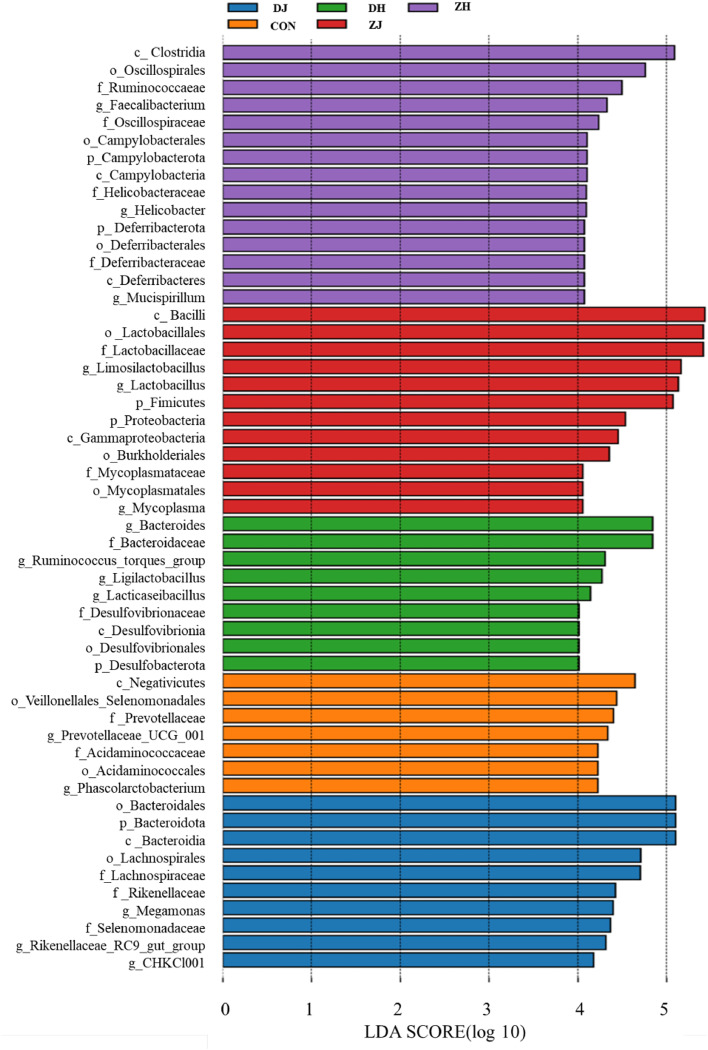
Bacterial taxa with linear discriminant analysis (LDA) that score greater than four in gut bacterial communities of laying hens feeding on different treatments. CON: basic diet, ZH: 5% of normal corn in the feed replaced with moldy corn, ZJ: 5% of normal corn replaced with moldy corn + 0.1 g/kg composite detoxification agent, DH: 5% of normal soybean meal replaced with moldy cottonseed meal, DJ: 5% of normal soybean meal replaced with moldy cottonseed meal + 0.1 g/kg composite detoxification agent.

**Fig. 7:**
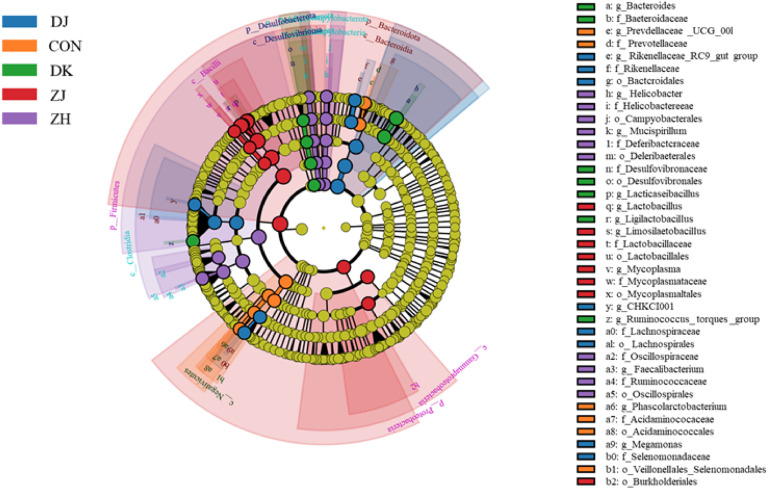
Cladogram of bacterial biomarkers from the phylum (innermost ring) to the genus (outermost ring) level, LDA score > 4. Differential bacterial taxa are marked with lowercase letters. The small circle of each different classification level represents the taxon of that level, and circle is proportional to relative abundance. The coloring principle is to use color the species. Those with no significant difference are yellow, while those of other different species are the highest species abundance. Different colors indicate different groups, and the nodes with different colors indicate communities that play an important of role in group represented by colors. CON: basic diet, ZH: 5% of normal corn in the feed replaced with moldy corn, ZJ: 5% of normal corn replaced with moldy corn + 0.1 g/kg composite detoxification agent, DH: 5% of normal soybean meal replaced with moldy cottonseed meal, DJ: 5% of normal soybean meal replaced with moldy cottonseed meal + 0.1 g/kg composite detoxification agent.

In order to identify the biomarkers that exhibited statistical differences between groups, we employed Linear Discriminant Analysis (LDA) and Least Effect Size (LEfSe) methods. Based on the condition that the LDA value was greater than 4 (as shown in Fig. 6), we screened various taxa (phyla, classes, orders, families, genera, and species) between different groups. Simultaneously, we constructed cladograms from phylum to genus to elucidate the distribution of various taxa at different taxonomic levels (Fig. 7). In the ZH, the gut microbiota of laying hen had the most different taxa (LDA>4). There were 15 taxa mainly concentrated in the Firmicutes, Campylobacterota, and Deferribacterota. In the ZJ, twelve different taxa differed in the gut microbiota of laying hen, of which nine belonged to the Firmicutes, and the other three belonged to the Proteobacteria. In the DH, nine different taxa differed in the gut microbiota of laying hen, of which two of the Bacteroidota, three of the Firmicutes, and four of the Desulfobacterota. In the DJ, ten different taxa differed in the gut microbiota of laying hen, of which five of the Bacteroidota and Firmicutes, respectively. In the CON, seven different taxa differed in the gut microbiota of laying hen, of which two of the Bacteroidota, five of the Firmicutes. Overall, these analyses demonstrated that ingestion had an effect on the gut community structure of laying hen were affected after consuming different treatment groups.

### Functional Prediction of Gut bacterial community

**Fig. 8:**
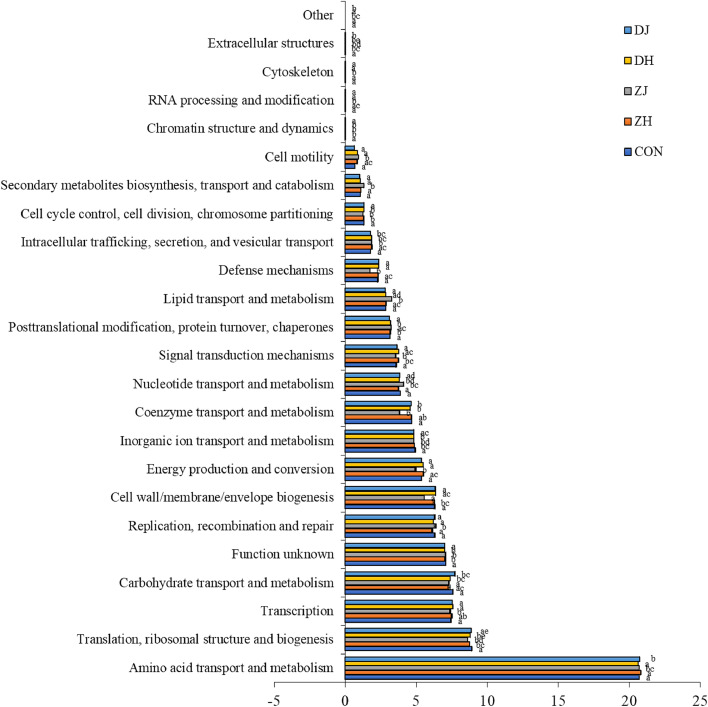
Comparison of predicted COG functions in gut bacterial communities of laying hens on different treatments. CON: basic diet, ZH: 5% of normal corn in the feed replaced with moldy corn, ZJ: 5% of normal corn replaced with moldy corn + 0.1 g/kg composite detoxification agent, DH: 5% of normal soybean meal replaced with moldy cottonseed meal, DJ: 5% of normal soybean meal replaced with moldy cottonseed meal + 0.1 g/kg composite detoxification agent.

In the present study, the metabolic potential of the gut bacterial community was predicted using PICRUSt2 (Fig. 8). The results indicated that the majority of predicted functional categories were predominantly associated with metabolic processes. The main metabolic functions include general function prediction only; amino acid transport and metabolism; translation, ribosomal structure and biogenesis; transcription; Carbohydrate transport and metabolism; function unknown; replication, recombination and repair; cell wall/membrane/envelope biogenesis; energy production and conversion; inorganic ion transport and metabolism; coenzyme transport and metabolism; nucleotide transport and metabolism; signal transduction mechanisms; posttranslational modification, protein turnover, chaperones; lipid transport and metabolism; defense mechanisms; intracellular trafficking, secretion, and vesicular transport. As the largest functional prediction category, ZJ groups proved to have the highest proportion of general function prediction only (11.84 ± 0.01%), Replication, recombination and repair (6.36 ± 0.04%), nucleotide transport and metabolism (4.12 ± 0.03%), posttranslational modification, protein turnover, chaperones (3.24 ± 0.01%), lipid transport and metabolism (3.28 ± 0.02%), secondary metabolites biosynthesis, transport and catabolism (1.31 ± 0.01%). ZH groups proved to have the highest proportion of amino acid transport and metabolism (9.12 ± 0.02%), energy production and conversion (5.51 ± 0.03%), signal transduction mechanisms (3.75 ± 0.02%), intracellular trafficking, secretion, and vesicular transport (1.88 ± 0.03%). CON groups proved to have the highest translation, ribosomal structure and biogenesis (8.91 ± 0.04%), carbohydrate transport and metabolism (7.60 ± 0.01%), function unknown (7.10 ± 0.02%), inorganic ion transport and metabolism (4.93 ± 0.02%), coenzyme transport and metabolism (4.68 ± 0.01%). DH groups proved to have the highest transcription (7.57 ± 0.01%), cell wall/membrane/envelope biogenesis (6.37 ± 0.01%). DJ groups proved to have the highest defense mechanisms (2.37 ± 0.01%).

### Relationships among of laying hen gut bacterial community and serum indices

The Spearman correlation results (Table 6) show that the diversity of the gut bacterial community associated with moldy feeds were significantly and positively associated with TP, BUN, T-AOC, MDA (*P* < 0.05) and CAT, GST (*P* < 0.01). The diversity of the gut bacterial community associated with moldy feeds were significantly and negatively associated with ALB, AST, GSH-Px (*P* < 0.05) and SOD, NO (*P* < 0.01). The diversity of the gut bacterial community associated with composite detoxification agent were significantly and negatively associated with TP, T-AOC, GST (*P* < 0.01). The diversity of the gut bacterial community associated with composite detoxification agent were positively associated with ALT (*P* < 0.05) and ALP, CAT, MDA (*P* < 0.01). The richness of the gut bacterial community associated with composite detoxification agent was positively associated with TP (*P* < 0.05) and T-AOC, GST (*P* < 0.01). The richness of the gut bacterial community associated with composite detoxification agent was negatively associated with GSH-Px (*P* < 0.05) and ALT, ALP, CAT, MDA (*P* < 0.01).

**Table 6:** Spearman correlations between alpha diversity of laying hens gut bacterial communities and serum indices.

Items	Moldy feeds		Composite detoxification agent	
	Shannon index	Chao1 index	Shannon index	Chao1 index
Total protein, TP (mg/mL)	0.650*	0.424	−0.777⁎⁎	0.693*
Albumin, ALB(mg/mL)	−0.636*	−0.495	0.283	0.113
Alanine aminotransferase, ALT(nmol/min/mg prot)	−0.721⁎⁎	−0.438	0.707*	−0.721⁎⁎
Aspartate aminotransferase, AST(nmol/min/mg prot)	−0.636*	−0.382	0.41	0.565
Alkaline phosphatase, ALP (nmol/min/mg prot)	−0.636*	−0.382	0.749⁎⁎	−0.834⁎⁎
Urea nitrogen, BUN (mg/mL)	0.636*	0.382	0.466	0.339
Total antioxidant capacity, T-AOC (μmol Trolox/mL)	0.636*	0.382	−0.749⁎⁎	0.806⁎⁎
Catalase, CAT (μmol/min/mg prot)	0.721⁎⁎	0.551	0.777⁎⁎	−0.721⁎⁎
Superoxide dismutase, SOD (U/mg prot)	−0.721⁎⁎	−0.438	−0.396	0.353
Glutathione peroxidase, GSH-Px (nmol/min/mg prot)	−0.636*	−0.382	0.537	−0.693*
glutathione S-transferase, GST (nmol/min/mg prot)	0.749⁎⁎	0.551	−0.749⁎⁎	0.806⁎⁎
Nitric oxide, NO (μmol/mL)	−0.721⁎⁎	0.551	0.269	0.099
Malondialdehyde, MDA (nmol/mL)	0.636*	0.382	0.749⁎⁎	−0.834⁎⁎

⁎ *P* < 0.05,.

⁎⁎ *P* < 0.01.

**Fig. 9:**
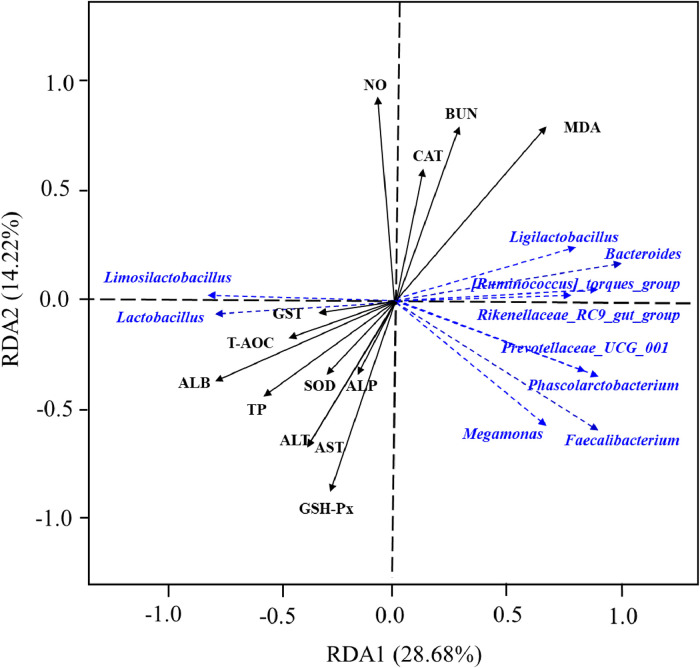
Redundancy analysis (RDA) of the relative abundance of laying hens gut bacterial communities and serum biochemical and antioxidant indicator. The blue dashed lines indicate laying hens gut bacterial community and black solid lines indicate grass nutrition composition. TP: Total protein, ALB: Albumin, ALT: Alanine aminotransferase, AST: Aspartate aminotransferase, ALP: Alkaline phosphatase, BUN: Urea nitrogen, T-AOC: Total antioxidant capacity, CAT: Catalase, SOD: Superoxide dismutase, GSH-Px: Glutathione peroxidase, GST: glutathione S-transferase, NO: Nitric oxide, MDA: Malondialdehyde. CON: basic diet, ZH: 5% of normal corn in the feed replaced with moldy corn, ZJ: 5% of normal corn replaced with moldy corn + 0.1 g/kg composite detoxification agent, DH: 5% of normal soybean meal replaced with moldy cottonseed meal, DJ: 5% of normal soybean meal replaced with moldy cottonseed meal + 0.1 g/kg composite detoxification agent.

The first and second axis of RDA explained 28.68% and 14.22% of the variance, respectively. The abundance of *Lactobacillus* and *Limosilactobacillus* were positively related to GST, T-AOC, ALB, TP, SOD, ALT, ALP, AST, and GSH-Px. The abundance of *Megamonas* and *Faecalibacterium* were positively related to ALT, ALP, AST, and GSH-Px. The abundance of *Prevotellaceae_UCG_001* and *Phascolarctobacterium* were positively related to GSH-Px. The abundance of *Rikenellaceae_RC9_gut_group* and *[Ruminococcus]_torques_group* were positively related to MDA, BUN, and CAT. *Bacteroides* and *Ligilactobacillus* were positively related to MDA, BUN, CAT, and NO (Fig. 9).

## Discussion

Dietary mycotoxins remain a persistent constraint to laying hen productivity and economic returns. In addition to the recognized toxicity of individual mycotoxins, concurrent exposure to multiple mycotoxins even at levels below current regulatory limits can produce synergistic effects, complicating both risk assessment and control strategies (Ma et al., 2024; Zhu et al., 2023). Therefore, investigations using naturally contaminated feed ingredients offer practical insights into subclinical risks and the field efficacy of dietary mitigation strategies.

In this study, hens fed diets containing mildly moldy corn and cottonseed meal exhibited reductions in egg weight and albumen quality, despite mycotoxin concentrations compliant with GB, 13078‑2017. These results are consistent with Zhang (2024), who reported minimal effects of low‑level contamination during early lay but emphasized cumulative risks under prolonged exposure. Fecal residue analysis provided mechanistic insight into the mode of action of a composite detoxifier. Although AFB₁ and ZEN were frequently below detection limits, extractable FB₁ was markedly reduced in the ZJ and DJ groups compared with ZH and DH, suggesting decreased intestinal bioaccessibility of FB₁. This shift in excretion coincided with improved laying performance and more favorable serum biochemical profiles. These findings highlight the potential of composite detoxifiers to mitigate subclinical mycotoxin impacts under production conditions.

Serum biochemistry further reflected treatment differences. Hens receiving ZJ and DJ maintained higher TP and ALB and showed modulated aminotransferase and alkaline phosphatase activities, indicative of suggestive of reduced hepatocellular stress (He et al., 2017; Marin et al., 2013; Samanc et al., 2011). In contrast, hens in the ZH group exhibited lower TP and ALB coupled with elevated blood urea nitrogen, consistent with mycotoxin induced disruption of hepatic synthesis and nitrogen metabolism (Zhu et al., 2023). These findings highlight the importance of integrating adsorbents, biotransformation enzymes, and antioxidants within a single formulation to effectively reduce toxin load and maintain metabolic homeostasis (Deníz et al., 2025; Gao et al., 2021; Guo et al., 2023).

Oxidative stress markers paralleled these biochemical trends. Moldy feed exposure reduced the activities of SOD, CAT, GPx, GST, and T-AOC while elevating MDA, aligning with the established role of mycotoxins in reactive oxygen species (ROS) overproduction (Ma et al., 2025; Surai et al., 2016). Composite detoxifier supplementation, particularly ZJ, mitigated these perturbations, supporting the view that reinforcement of endogenous antioxidant defenses is central to counteracting mycotoxin toxicity (Chen et al., 2019; Sorrenti et al., 2013).

Gut microbiota analyses revealed treatment specific restructuring of the microbial community. The DH group displayed higher Shannon and Simpson indices, whereas ZJ increased ACE and Chao1 metrics, indicating altered richness. At the phylum level, Proteobacteria were enriched in ZJ, while Firmicutes and Bacteroidota predominated in CON, ZH, DH, and DJ. At the genus level, ZJ selectively promoted *Lactobacillus* and *Limosilactobacillus*, taxa linked to gut homeostasis and short chain fatty acid production (Azad et al., 2018). Predicted KEGG pathways suggested enhanced amino acid and carbohydrate metabolism in CON, upregulated replication and repair functions in ZJ, and enriched defense mechanisms in DJ. While these functional predictions imply a microbiome better poised to cope with xenobiotic and oxidative challenges, direct evidence from metagenomics or metatranscriptomics is required to substantiate these inferences.

Correlation analyses highlighted complex microbiota–host linkages under mycotoxin challenge. In moldy feed groups, taxa such as *Rikenellaceae_RC9_gut_group* and *[Ruminococcus]_torques_group* correlated positively with MDA and BUN, potentially reflecting dysbiosis secondary to mucosal impairment (Ma et al., 2024). Conversely, in ZJ treated hens, beneficial genera including *Lactobacillus, Limosilactobacillus, Megamonas*, and *Faecalibacterium* were positively associated with TP, ALB, SOD, and GSH-Px. These observations suggest that the composite detoxifier may foster a microbial milieu conducive to antioxidant defense and metabolic stability. Nevertheless, because intestinal morphology and barrier function were not directly measured, assertions regarding restored barrier integrity remain hypothetical. Further studies employing germ free models or fecal microbiota transplantation are warranted to clarify whether these microbial shifts causally mediate the observed physiological benefits.

## Conclusions

In this study, the composite detoxification agents partially alleviated adverse effects of moldy feed on laying performance, egg quality, serum biochemical profiles, and antioxidant status. Observed shifts in gut microbiota composition were closely associated with these physiological improvements; enrichment of beneficial genera correlated with better antioxidant and hepatic synthetic markers, whereas dysbiotic taxa tracked with damage indicators. These findings suggest that the protective effects are associated with, and may be partly explained by, modulation of the gut microbiota-host axis. However, direct mechanistic evidence for a causal role of the microbiota in detoxification requires further investigation.

## Funding

This study was supported by the Youth Fund Project of Gansu Academy of Sciences (Project Number: 2024QN-16).

## CRediT authorship contribution statement

**Yaling Ma:** Writing – review & editing, Writing – original draft, Project administration, Funding acquisition. **Qiaomei Gao:** Supervision, Resources, Methodology, Data curation. **Junai Lu:** Writing – original draft, Software, Methodology, Formal analysis. **Xianbai Liu:** Writing – review & editing, Supervision, Resources, Conceptualization.

## Disclosures

The authors declare no conflict of interests.

## Appendix Supplementary materials

Image, application 1

Image, application 2

## Footnotes

Supplementary material associated with this article can be found, in the online version, at doi:10.1016/j.psj.2026.107347.
