# Supplementary material for: The composite detoxification agent alleviates the toxicity induced by mycotoxins in Hy-Line Brown laying hens by regulating antioxidant capacity and gut bacterial communities
Source: Poult Sci. 2026 Jun 26;105(10):107347. doi: 10.1016/j.psj.2026.107347 (PMC13356725; doi:10.1016/j.psj.2026.107347)
Supplement: Supplementary file 1 [file mmc1.pdf]

兰州大学草地农业科技学院伦理委员会
伦理审查批准表
Ethics Committee of College of Pastoral Agriculture Science and Technology of Lanzhou University
Ethics Review Approval Form

课题名称：真菌毒素损伤动物肠道屏障功能机理研究<br>Project title: Research on the mechanism of mycotoxin damage to the intestinal barrier function of animals		
课题负责人：王虎成<br>Project leader: Hucheng Wang	职称：教授<br>Professional Title: Associate professor	联系方式：13619362065<br>Tel: 13619362065
研究单位：兰州大学草地农业科技学院<br>Department: College of Pastoral Agriculture Science and Technology, Lanzhou University		
研究课题来源：<br>Project source:	☑政府 ☐基金会 ☐企业 ☐国际合作 ☐自主 ☐其他<br>☑Government ☐NGO Foundation ☐Enterprise ☐International cooperation ☐Independence ☐Others	

审查意见：
Review comment:

王虎成老师的研究方案经伦理委员会审查：
Under the review of Ethics committee, the research procedure of Hucheng Wang:

☑ 符合伦理学要求，同意按照此方案进行研究。
in accord with the ethic requirements from Lanzhou university Ethics Committee, agree to study.

☐ 研究方案修改或补充资料后，伦理委员会同意开始研究。
will be agree after proposal modification and supplement of more files.

☐ 不符合伦理要求，请修改后报伦理委员会再审查。
unqualified the ethics requirement, need to submit the application to further review after modification.

☐ 终止或暂停已批准的研究。
totally unsuited to ethics requirements, need to stop research.

兰州大学草地农业科技学院伦理学委员会
Ethics Committee of College of Pastoral Agriculture Science and Technology of Lanzhou University
草地农业科技学院代章
Stamp of College of Pastoral Agriculture Science and Technology
2023 年 9 月 20 日
Sep 20th, 2023
