# Supplementary material for: The composite detoxification agent alleviates the toxicity induced by mycotoxins in Hy-Line Brown laying hens by regulating antioxidant capacity and gut bacterial communities
Source: Poult Sci. 2026 Jun 26;105(10):107347. doi: 10.1016/j.psj.2026.107347 (PMC13356725; doi:10.1016/j.psj.2026.107347)
Supplement: Supplementary file 2 [file mmc2.docx]

**Table S1.** Concentrations of mycotoxins in artificially molded ingredients (air-dry basis)

| Items, *μ*g/kg | Moldy corn | Moldy cottonseed meal | Mycotoxin standard of limit, (GB 13078-2017, China) |
| --- | --- | --- | --- |
| Aflatoxins B_1_ | 80.83 | 78.09 | 20 |
| Deoxynivalenol | 415.77 | 2 982.85 | 3000 |
| Zearalenone | 148.36 | 602.17 | 500 |
| Fumonisins B_1_ | 48964.04 | 26974.25 | 20000 |
| Ochratoxin A | <10 | <10 | 100 |
| T-2 toxin | <4 | <4 | 500 |

**Table S2.** Composition and nutrient levels of basal diets (air-dry basis) %

| Items | Content |
| --- | --- |
| Ingredients |  |
| Corn | 62.00 |
| Soybean meal | 27.00 |
| Soybean oil | 1.10 |
| Limestone | 8.00 |
| NaCl | 0.20 |
| CaHPO_4_ | 1.50 |
| ^1^Premix | 0.20 |
| Total | 100.00 |
| Nutrient levels |  |
| ^2^ME (MJ/kg) | 11.58 |
| CP，% | 16.48 |
| Ca | 3.51 |
| TP | 0.67 |

^1^ The premix provided the following per kg of diet: Vitamin D_3_ (VD_3_) 2 500 IU, Vitamin A (VA) 9 500 IU, Vitamin E (VE) 30 IU, Vitamin K_3_ (VK_3_) 2.65 mg, Vitamin B_12_ (VB_12_) 0.025 mg, Vitamin B_2_ (VB_2_) 6 mg, Pantothenic acid 12 mg, Biotin 0.032 5 mg, Folic acid 1.25 mg, Ferrous sulfate (Fe）80 mg, Sodium selenite (Se）0.15 mg, Nicotinic acid 50 mg, Manganese sulfate (Mn) 100 mg, Calcium iodate (I) 0.35 mg, Zinc sulfate (Zn) 75 mg, Copper sulphate (Cu) 8 mg.

^2^ ME was a calculated value, while the others were measured values.

**Table S3.** Formula for the composite detoxification agent

| Items | Viable bacterial count | Company | Dosage, g/kg |
| --- | --- | --- | --- |
| *Lactobacillus plantarum* | 100 billion/kg | Kangfengyuan Biotechnology Co., Ltd., China | 0.6 |
| Mannose-oligosaccharide | / | Xuzhou Saifu Biotechnology Co., Ltd., China | 1 |
| Montmorillonite | / | Qsllour Biotechnology Co., Ltd., Chaina | 0.5 |

**Table S4.** Detection results of mycotoxins in feeding different treatments (air-dry basis)

| Items, *μ*g/kg | CON | ZH | ZJ | DH | DJ | Mycotoxin standard of limit, (GB 13078-2017, China) |
| --- | --- | --- | --- | --- | --- | --- |
| Aflatoxins B_1_ | 4.89 | 13.94 | 6.16 | 8.91 | 5.75 | 20 |
| Deoxynivalenol | <100 | <100 | <100 | 141.27 | <100 | 3000 |
| Zearalenone | 10.85 | 68.36 | 21.09 | 93.42 | 40.43 | 500 |
| Fumonisins B_1_ | <100 | 14385.65 | 7601.02 | 13844.39 | 6335.44 | 20000 |
| Ochratoxin A | <10 | <10 | <10 | <10 | <10 | 100 |
| T-2 toxin | <4 | <4 | <4 | <4 | <4 | 500 |

CON: basic diet, ZH: 5% of normal corn in the feed replaced with moldy corn, ZJ: 5% of normal corn replaced with moldy corn + 0.1 g/kg composite detoxification agent, DH: 5% of normal soybean meal replaced with moldy cottonseed meal, DJ: 5% of normal soybean meal replaced with moldy cottonseed meal + 0.1 g/kg composite detoxification agent.

**Table S5.** Detection results of mycotoxins in fecal different treatments (air-dry basis)

| Items, *μ*g/kg | CON | ZH | ZJ | DH | DJ | Mycotoxin standard of limit, (GB 13078-2017, China) |
| --- | --- | --- | --- | --- | --- | --- |
| Aflatoxins B_1_ | <4 | 12.44 | <4 | 8.04 | <4 | 20 |
| Deoxynivalenol | <100 | <100 | <100 | 132.44 | <100 | 3000 |
| Zearalenone | <10 | 64.19 | 14.91 | 90.21 | 32.98 | 500 |
| Fumonisins B_1_ | <100 | 12345.74 | 936.56 | 13377.18 | 1117.53 | 20000 |
| Ochratoxin A | <10 | <10 | <10 | <10 | <10 | 100 |
| T-2 toxin | <4 | <4 | <4 | <4 | <4 | 500 |

CON: basic diet, ZH: 5% of normal corn in the feed replaced with moldy corn, ZJ: 5% of normal corn replaced with moldy corn + 0.1 g/kg composite detoxification agent, DH: 5% of normal soybean meal replaced with moldy cottonseed meal, DJ: 5% of normal soybean meal replaced with moldy cottonseed meal + 0.1 g/kg composite detoxification agent.

**Table S6.** Summary of sequence statistics for the PacBio runs of all samples.

| Sample ID^1^ | Raw CCS^2^ | Clean CCS | Effective CCS | AvgLen (bp) | Effective (%) |
| --- | --- | --- | --- | --- | --- |
| CON1 | 66971 | 66838 | 65928 | 1463 | 98.44 |
| CON2 | 61652 | 61547 | 60949 | 1465 | 98.86 |
| CON3 | 65113 | 64950 | 64390 | 1465 | 98.89 |
| CON4 | 55729 | 55611 | 54994 | 1465 | 98.68 |
| CON5 | 58297 | 58191 | 57698 | 1464 | 98.97 |
| CON6 | 66567 | 66418 | 65860 | 1464 | 98.94 |
| ZH1 | 55778 | 55625 | 54755 | 1459 | 98.17 |
| ZH2 | 62884 | 62760 | 62194 | 1461 | 98.9 |
| ZH3 | 62596 | 62497 | 61857 | 1461 | 98.82 |
| ZH4 | 58416 | 58292 | 57337 | 1462 | 98.15 |
| ZH5 | 64451 | 64350 | 63979 | 1462 | 99.27 |
| ZH6 | 54003 | 53940 | 53487 | 1462 | 99.04 |
| DH1 | 61911 | 61771 | 61194 | 1462 | 98.84 |
| DH2 | 58386 | 58254 | 57678 | 1462 | 98.79 |
| DH3 | 65054 | 64865 | 64249 | 1462 | 98.76 |
| DH4 | 66926 | 66740 | 66035 | 1462 | 98.67 |
| DH5 | 56745 | 56569 | 56149 | 1462 | 98.95 |
| DH6 | 59349 | 59223 | 58669 | 1462 | 98.85 |
| ZJ1 | 66921 | 66902 | 63785 | 1477 | 95.31 |
| ZJ2 | 59380 | 59368 | 56839 | 1478 | 95.72 |
| ZJ3 | 61539 | 61525 | 59183 | 1481 | 96.17 |
| ZJ4 | 64974 | 64960 | 62026 | 1475 | 95.46 |
| ZJ5 | 63622 | 63596 | 61158 | 1479 | 96.13 |
| ZJ6 | 64570 | 64542 | 62557 | 1480 | 96.88 |
| DJ1 | 59286 | 59169 | 57954 | 1466 | 97.75 |
| DJ2 | 58179 | 58085 | 56015 | 1465 | 96.28 |
| DJ3 | 65953 | 65839 | 64671 | 1466 | 98.06 |
| DJ4 | 54721 | 54628 | 52985 | 1466 | 96.83 |
| DJ5 | 54929 | 54819 | 53903 | 1466 | 98.13 |
| DJ6 | 61911 | 61760 | 60575 | 1466 | 97.84 |

^1^ CON: basic diet, ZH: 5% of normal corn in the feed replaced with moldy corn, ZJ: 5% of normal corn replaced with moldy corn + 0.1 g/kg composite detoxification agent, DH: 5% of normal soybean meal replaced with moldy cottonseed meal, DJ: 5% of normal soybean meal replaced with moldy cottonseed meal + 0.1 g/kg composite detoxification agent.

^2^CCS, Circular Consensus Sequencing.

**Table S7.** Summary of classification of the laying hens species based on sequencing of 16S rRNA gene amplicons.

| Sample ID^1^ | Phylum | Class | Order | Family | Genus | Species |
| --- | --- | --- | --- | --- | --- | --- |
| DJ1 | 20 | 35 | 82 | 144 | 275 | 414 |
| DJ2 | 22 | 40 | 102 | 181 | 362 | 578 |
| DJ3 | 22 | 41 | 104 | 174 | 332 | 499 |
| DJ4 | 22 | 42 | 101 | 194 | 397 | 601 |
| DJ5 | 25 | 45 | 100 | 177 | 337 | 508 |
| DJ6 | 19 | 29 | 71 | 135 | 266 | 405 |
| CON1 | 27 | 52 | 118 | 203 | 418 | 637 |
| CON2 | 25 | 45 | 97 | 162 | 344 | 529 |
| CON3 | 22 | 40 | 91 | 158 | 341 | 520 |
| CON4 | 25 | 43 | 92 | 159 | 335 | 536 |
| CON5 | 26 | 48 | 108 | 189 | 384 | 579 |
| CON6 | 24 | 43 | 96 | 165 | 347 | 553 |
| DH1 | 28 | 51 | 113 | 199 | 410 | 619 |
| DH2 | 27 | 52 | 122 | 217 | 434 | 663 |
| DH3 | 29 | 54 | 122 | 214 | 428 | 654 |
| DH4 | 29 | 57 | 131 | 231 | 463 | 708 |
| DH5 | 30 | 53 | 110 | 194 | 393 | 606 |
| DH6 | 29 | 54 | 120 | 216 | 433 | 652 |
| ZJ1 | 32 | 62 | 146 | 256 | 506 | 793 |
| ZJ2 | 30 | 60 | 147 | 270 | 540 | 840 |
| ZJ3 | 30 | 61 | 142 | 257 | 498 | 780 |
| ZJ4 | 28 | 61 | 143 | 254 | 507 | 832 |
| ZJ5 | 32 | 64 | 152 | 283 | 538 | 845 |
| ZJ6 | 30 | 62 | 154 | 274 | 530 | 829 |
| ZH1 | 26 | 54 | 131 | 234 | 486 | 726 |
| ZH2 | 24 | 51 | 109 | 201 | 421 | 587 |
| ZH3 | 28 | 59 | 129 | 230 | 451 | 638 |
| ZH4 | 25 | 50 | 117 | 216 | 442 | 621 |
| ZH5 | 25 | 52 | 105 | 196 | 405 | 571 |
| ZH6 | 25 | 51 | 109 | 197 | 399 | 564 |
| Total | 37 | 82 | 209 | 397 | 918 | 1620 |

^1^ CON: basic diet, ZH: 5% of normal corn in the feed replaced with moldy corn, ZJ: 5% of normal corn replaced with moldy corn + 0.1 g/kg composite detoxification agent, DH: 5% of normal soybean meal replaced with moldy cottonseed meal, DJ: 5% of normal soybean meal replaced with moldy cottonseed meal + 0.1 g/kg composite detoxification agent.


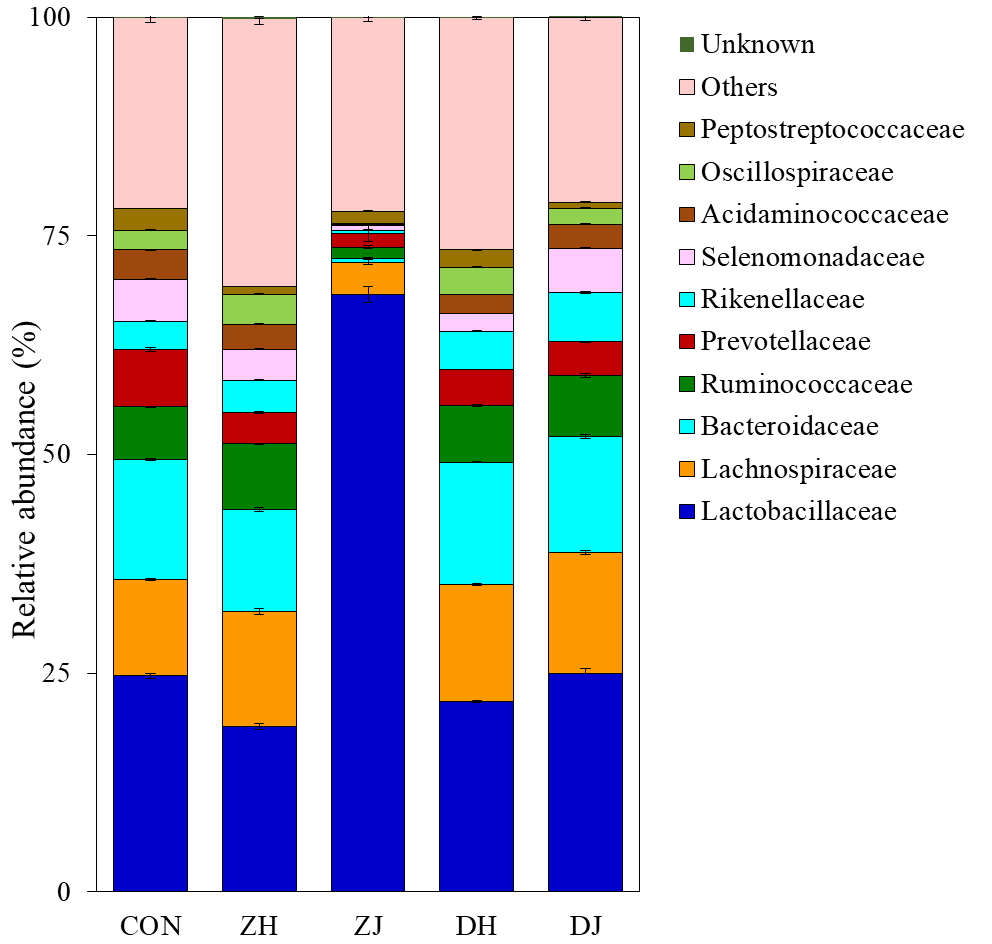


**Figure S1.** The top 10 relative abundances of gut bacterial communities at family level. Each color indicate a species, the height of the color block represents the proportion of species in relative abundance. The other species shown in figure are merged into “other”. “Unknown” indicates species that there are not received a taxonomic annotation. CON: basic diet, ZH: 5% of normal corn in the feed replaced with moldy corn, ZJ: 5% of normal corn replaced with moldy corn + 0.1 g/kg composite detoxification agent, DH: 5% of normal soybean meal replaced with moldy cottonseed meal, DJ: 5% of normal soybean meal replaced with moldy cottonseed meal + 0.1 g/kg composite detoxification agent.
